# Late onset Alzheimer’s Disease mouse model with altered synaptic and behavior phenotypes

**DOI:** 10.1002/alz.094617

**Published:** 2025-01-09

**Authors:** Ankit Seth, Jacob Bradford Miller, Ashiq M. Rafiq, Ferenc Deak

**Affiliations:** ^1^ Augusta University, Augusta, GA USA

## Abstract

**Background:**

Genetically modified mouse models have provided a vital understanding of the Alzheimer’s disease (AD) mechanisms, but these models were generally focused on familial AD and do not recapitulate the late‐onset human AD pathophysiology. To circumvent the complications of existing AD mice models, we are investigating a novel non‐mutant humanized amyloid beta (Aβ) expressing mouse line that expresses the humanized mouse App gene within the Aβ peptide sequence

**Method:**

This study aims to investigate the learning and memory function in this novel human Aβ knock‐in (h‐Aβ KI) mice of different age and sex groups using behavioral tests, neuronal circuitry, and long‐term potentiation experiments.

**Result:**

Behavioral studies revealed age and sex specific phenotype: 12 and 18 months old female h‐Aβ KI mice demonstrated reduced locomotor and exploration activities along with pronounced deficits in the preference for social novelty. h‐Aβ KI mice also showed impaired spatial learning and memory in behavior tests as well as reduced synaptic plasticity in hippocampal CA1 and DG regions. Cultured h‐Aβ KI hippocampal neurons had significantly slower rate of synaptic vesicle release but no difference in average recycling pool sizes compared to wild‐type control neurons in fluorescent FM1‐43 assays. Live imaging of neurons loaded with FM2‐10 dye revealed differences in synaptic release dynamics of readily‐releasable pool between female and male h‐Aβ KI mice with sucrose stimulation. Immunohistology revealed no amyloid deposit at these age and needs further investigation at older ages of this animal model.

**Conclusion:**

Our data demonstrate that expression of wild‐type human Aβ in mice is sufficient to drive synaptic and cognitive changes during aging without plaque deposition.

